# Seventy years’ achievements of international cooperation by the National Institute of Parasitic Diseases at the Chinese Center for Disease Control and Prevention

**DOI:** 10.1186/s40249-020-00783-3

**Published:** 2020-11-30

**Authors:** Ru-Bo Wang, Yi-Ting Hong, Xiao-Nong Zhou

**Affiliations:** National Institute of Parasitic Diseases, Chinese Center for Disease Control and Prevention; Chinese Center for Tropical Diseases Research; WHO Collaborating Centre for Tropical Diseases; National Center for International Research on Tropical Diseases, Ministry of Science and Technology; Key Laboratory of Parasite and Vector Biology, Ministry of Health, Shanghai, China

**Keywords:** Seventy years, International cooperation, The National Institute of Parasitic Diseases

## Abstract

**Background:**

With the promotion of national control programs on parasitic and tropical diseases in China, the National Institute of Parasitic Diseases (NIPD), Chinese Center for Disease Control and Prevention has gained significant experience in the global health arena through international cooperation over the last seven decades allowing a multilateral impact in the elimination of major endemic diseases.

**Methods:**

The achievements of NIPD since 1950 has been analyzed with emphasis on the various stages that started with research and control of the endemic parasitic and other tropical diseases at the national level and progressed via international cooperation into a global presence.

**Results:**

The major achievements contributed by NIPD consist of (i) improving technical capability; (ii) promoting control and elimination of parasitic and tropical diseases; (iii) participating in global health governance and cooperation; and (iv) developing a cooperation model for technical assistance and global public health development. It is expected that NIPD’s experience of international cooperation will be essential for the dissemination of China's successful experience in global health governance, emergency response and development, with focus on malaria and neglected tropical diseases such as schistosomiasis, soil-borne and food-borne helminthiases and echinococcosis.

**Conclusions:**

NIPD’s new tasks will not only continue to promote national control of endemic parasitic infections and disease elimination programs in China, but also play a leading role in global health and disease elimination programs in the future.

## Background

Established in 1950, the National Institute of Parasitic Diseases (NIPD, the former name was the Huadong Branch of the National Institute of Health in 1950) is a state-level institution affiliated with the Chinese Center for Disease Control and Prevention (China CDC), with its main tasks of control, research and service with respect to parasitic, and tropical diseases [1]. The year 2020 is the 70th anniversary for NIPD’s achievements over seven decades of national research and control on tropical diseases, which has progressed into large-scale international cooperation promoting the development of multi-lateral cooperation, participation in global health governance and transferring China's experience in the control of parasitic diseases abroad. Together with its focus on training of staff and promotion of national control programs on parasitic and other tropical diseases, NIPD has gained significant presence the world over in global health programs, particularly in Southeast Asia and Africa.

## History

NIPD seventy years’ achievements on international cooperation have been impacted across different periods reflected in three stages.

### Exploring cooperation stage (1950–1977)

During this stage, NIPD initiated and carried out a number of international cooperation activities besides its national responsibilities. This was done to get a preliminary understanding of the international scientific research on parasitic diseases and to create bridges and a stable exchange with the World Health Organization (WHO). In the 1950s, the international cooperation was not yet prominent, mainly including the reception of scholars or officials from neighbouring countries such as the former Soviet Union, Japan, Vietnam, etc. Later, in the 1960s and early 1970s, the international cooperation was slowed down, with only few international experts visiting NIPD. From 1955 to 1964, NIPD had visitors from 28 countries but from 1965 to 1977 only nine. During these years, there were only four overseas visits by NIPD staff and one international conference on parasitic diseases held in China, including the National Academic Conference on Parasitic Diseases held in Shanghai in 1958 with participants from five countries.

### Resuming development stage (1978–2010)

With the implementation of economic reform and the opening policy in China, the international cooperation were resumed developing at a rapid pace. Every year of that period, nearly 30 groups (40 person-times) from NIPD paid visits abroad and more than 30 groups (100 person-times) visited NIPD. International conferences and training courses were held and NIPD carried out international cooperative projects. In 1979, NIPD was authorized to establish the WHO Collaborating Center for Malaria, Schistosomiasis and Filariasis [2], which led to increased cooperation and frequent WHO exchange. In 1978, Dr. Thomas Adeoye Lambo, then Deputy Director-General of WHO, visited followed 1998 by Dr. Gro Harlem Brundtland, then Director-General of WHO. From 1980 to 1985, the United Nations International Children's Emergency Fund (UNICEF)/the United Nations Development Programme (UNDP)/World Bank/WHO Special Programme for Research and Training in Tropical Diseases (TDR) provided the NIPD with an institutional strengthening fund that led to large-scale research on parasitic diseases. A total of 11 NIPD experts have served as members of different WHO Expert Committees.

### Sharing development stage (2011–2020)

The international cooperation of NIPD expanded from parasitic diseases to general tropical diseases and global health cooperation for the purpose of building “an international cooperation centre for tropical diseases with the functions of disease control, research, policy consultation, education and training and global health”. In 2014, the “International Joint Research Center for Tropical Diseases” was established at NIPD by the Ministry of Science and Technology of China, and in 2017, the new Chinese Center for Tropical Diseases Research (CTDR) was designated for NIPD by the Chinese Government, with its functions extended into the tropical disease research. Every year, more than 40 groups (80 person-times) paid visits abroad, and more than 110 person-times called in on NIPD. In 2011, Dr. Margaret Chan, then Director-General of WHO, paid a visit to NIPD unveiling plans for redesigning the WHO Collaborating Center for Malaria, Schistosomiasis and Filariasis at NIPD into the WHO Collaborating Centre for Tropical Diseases that took place 2015.

The NIPD has broadened its international partnerships by signing cooperation Memoranda of Understanding (MOU)-based cooperation with 13 international organizations (e.g. the Swiss Tropical and Public Health Institute, the London School of Hygiene & Tropical Medicine), and establishing five international cooperation networks including the Regional Network on Asian Schistosomiasis and Other Helminth Zoonoses (RNAS+) in1998, the Institutional-Based Network of China-Africa Cooperation on Schistosomiasis Elimination (INCAS) in 2015, the Asia–Pacific Network on Drug and Diagnostics Innovation (AP-NDI) in 2017, the Belt and Road Network for the Elimination and Control of Echinococcosis and Cysticercosis (BR-NEC) in2017 and the Institutional-based Network of China-Africa Cooperation on Malaria Elimination (INCAM) in 2018. Since 2012, NIPD has held five international symposia on surveillance-response systems aiming eliminate various tropical diseases and launched *Infectious Diseases of Poverty*, an international peer review journal in 2012.

## Achievements

### Improving NIPD’s technical capability

Extensive international exchange has cultivated national academic leaders, strengthened talent teams and promoted the building of parasitological research capabilities. For example, under the support of the TDR institutional strengthening fund, more than 10 researchers could extend studies abroad, enter cooperative research, embark on Doctor of Philosophy Degree(PhD), carrying out cooperative research on diagnostic reagents, vaccines and drugs to become academic leaders. Through the implementation of the World Bank Loan Project (WBLP) for Schistosomiasis Control in China (1992–2001) [3], the China Global Fund Malaria Program (2003–2012) and the China-UK Global Health Support Projects, a group of researchers, managers and global health staff have received training with the focus on controlling endemic, parasitic diseases, thereby enhancing the technical capability to international levels.

### Promoting control and elimination of parasitic diseases in China

With the leadership of the Ministry of Health/the National Health Commission of China, NIPD participated in the application and the implementation of international health cooperation projects through active cooperation with international organizations such as WHO, the World Bank, the Global Fund to Fight AIDS, Tuberculosis and Malaria, and others. Besides covering the gaps in disease control, new ideas were advanced, new concepts conceived, new technologies and health products introduced, which together promoted the control and elimination of parasitic diseases in China. Through implementation of WBLP, the number of schistosomiasis patients decreased by close to 50%, and the positive rate of faecal examination of cattle decreased by more than this [3].Under the support of China's Global Fund Malaria Control Program, close to 12 million patients were diagnosed, about 1.5 million cases were treated and more than 1.5 million long-lasting insecticidal nets were distributed. These actions led to a decrease of the malaria incidence from four per 10 000 population in 2003 to below one per 10 000 in 2012 promoting the goal in the fight against malaria in China from control to elimination [4].

### Participating in global health governance and cooperation

As a WHO Collaborating Centre, NIPD has so far provided 11 members of the WHO Expert Committee and sent additional technical experts to WHO headquarters as well as its China and Africa regional offices. NIPD sends staffs on an annual basis participating in the WHO Technical Conference on Schistosomiasis, Malaria and Neglected Tropical Diseases, contributing to agenda setting, program formulation, technical guideline drafting and rule-making for global parasitic and tropical diseases. NIPD also provides technical consultation for the global control of parasitic and tropical diseases. China’s strategy for the control/elimination of filariasis and schistosomiasis, including the use of diethylcarbamazine medicated salt for filariasis control, surveillance/response systems for interrupting the transmission of filariasis and schistosomiasis have been elevated to reference documents in WHO’s technical programmes. NIPD has actively participated in the China-UK health discussions and the international health dialogue between Brazil, Russia, India and China (BRIC). NIPD has in addition established regional multilateral cooperation network on tropical diseases and is holding a biannual series of international symposia on surveillance-response systems leading to tropical diseases elimination, to exchange to share information about the control of parasitic and tropical diseases.

### Exploring the cooperation model for technical assistance and public health development

From 2007 to 2009, NIPD participated in compiling the guideline for assistance in the construction of 30 malaria control centres in Africa, and sent three experts of malaria control to Cameroon, Chad and other countries for on-site operative activities. Since 2013, the schistosomiasis and malaria control assistance in Africa was listed as the priority project in China-Africa cooperation [5]. Since 2015, NIPD had launched a new model of tripartite cooperation, including the China-UK-Tanzania Pilot Project on Malaria Control [6] and the Australia-China-Papua New Guinea Trilateral Development Cooperation Project on Malaria Control, so as to assist Tanzania and Papua New Guinea strengthening malaria control. NIPD also explores the applicability of Chinese experience, technology or products to many other countries, e.g., experts were sent to Sierra Leone in the 2014–2020 period, to control and eventually eliminate Ebola transmission there and establish China-Sierra Leone technology cooperation resulting in a Bio safety Level III Fixed Laboratory. In addition, NIPD successfully carried out the research projects on new sustainable strategies for schistosomiasis control and elimination and other major parasitic diseases in six Asian countries promoting the development of research cooperative projects on the control of parasitic diseases there. Since 2014, the NIPD has held four China-Myanmar seminars on malaria control to promote joint prevention and control of malaria at the China-Myanmar border area and participated in the joint control of malaria in the Lancang-Mekong region carried out by China and five other countries to deepen the “Belt and Road” health cooperation [7].

## Prospects

With the concept of a community with a shared future for mankind, under the framework of cooperation of “Belt and Road Initiative” and China-Africa Cooperation Forum, NIPD aims to integrate available resources, strengthen capacity-building, and expand cooperation with other countries and international organizations [8]. In the future, the international cooperation strategy of NIPD is designed under the following headlines:

1. Vision: promoting the move from controlling parasitic and tropical diseases to their worldwide elimination; to become a more active participant in global health cooperation, global health governance and global health emergency response; and to disseminate China's successful experience to other endemic countries.
2. Cooperating regions: covering the world with special reference to Africa, Southeast Asia, Central Asia, Oceania and Latin America.
3. Diseases: focusing on malaria and all neglected tropical diseases, with special focus on schistosomiasis, soil-borne/food-borne helminthiasis and echinococcosis.
4. Platform: WHO Collaborating Centre for Tropical Diseases at NIPD, the international cooperation networks (RNAS+, INCAS, AP-NDI, BR-NEC, and INCAM), the international symposium on surveillance-response systems leading to tropical disease elimination.
5. Activities: international academic exchanges, international talent cooperation and training, research and pilot projects in Africa, Southeast Asia and the establishment of overseas tropical disease control and research bases in Asia and Africa.

## Conclusions

NIPD’s seven decades of continuous achievements should should be the leading light in encouraging the young generation to wholeheartedly engage in governance development and security of global health. Through the existing networks cosponsored by NIPD, activities should increasingly be performed together with colleagues from Africa and Southeast Asia through the establishment of overseas tropical disease control projects and/or research bases, in order to achieve the vision of NIPD promoting control/elimination of tropical diseases worldwide by disseminating China’s successful experience.

## Data Availability

Not applicable.

## Abbreviations

NIPD: National Institute of Parasitic Diseases at China CDC
China CDC: Chinese Center for Disease Control and Prevention
WHO: World Health Organization
TDR: UNICEF/UNDP/World Bank/WHO Special Programme for Research and Training in Tropical Diseases
CTDR: Chinese Center for Tropical Diseases Research
RNAS+: Regional Network on Asian Schistosomiasis and Other Helminth Zoonoses
INCAS: Institutional-Based Network of China-Africa Cooperation on Schistosomiasis Elimination
AP-NDI: Asia–Pacific Network on Drug and Diagnostics Innovation
BR-NEC: B&R Network for the Elimination and Control of Echinococcosis and Cysticercosis
INCAM: Institutional-based Network of China-Africa Cooperation on Malaria Elimination
BRICs: Federative Republic of Brazil, Russian Federation, Republic of India, People's Republic of China and Republic of South Africa

## References

[1] Yu SH, Zhou XN (2010). Records of national institute of parasitic diseases, China CDC (1950–2010).

[2] Su JJ (2018). The commence of multilateral health diplomacy of the People’s Republic of China. Chin J Hist Sci Technol.

[3] Chen XY, Wang LY, Cai JM, Zhou XN, Zheng J, Guo JG (2005). Schistosomiasis control in China: the impact of a 10-year World Bank Loan Project (1992–2001). Bull World Health Organ..

[4] Wang RB, Zhang QF, Zheng B, Xia ZG, Zhou SS, Tang LH (2014). Transition from control to elimination: impact of the 10-year global fund project on malaria control and elimination in China. Adv Parasitol..

[5] Xu J, Yu Q, Tchuente LA, Bergquist R, Sacko M, Utzinger J (2016). Enhancing collaboration between China and African countries for schistosomiasis control. Lancet Infect Dis.

[6] Wang DQ, Chaki P, Mlacha Y, Gavana T, Michael MG, Khatibu R (2019). Application of community-based and integrated strategy to reduce malaria disease burden in southern Tanzania: the study protocol of China-UK-Tanzania pilot project on malaria control. Infect Dis Poverty.

[7] Chen J, Bergquist R, Zhou XN, Xue JB, Qian MB (2019). Combating infectious disease epidemics through China's belt and road initiative. PLoS Neglected Trop Dis..

[8] Guan Y, Xiao N, Zhou XN (2020). The role of the WHO Collaborating Centre for Tropical Diseases in China. China Wkly.

